# Advancing improvement in riverine water quality caused a non-native fish species invasion and native fish fauna recovery

**DOI:** 10.1038/s41598-021-93751-2

**Published:** 2021-08-13

**Authors:** Łukasz Głowacki, Andrzej Kruk, Tadeusz Penczak

**Affiliations:** Department of Ecology and Vertebrate Zoology, Faculty of Biology and Environmental Protection, University of Lodz, 12/16 Banacha Str., 90-237 Łódź, Poland

**Keywords:** Community ecology, Freshwater ecology, Invasive species, Population dynamics, Restoration ecology

## Abstract

The knowledge of biotic and abiotic drivers that put non-native invasive fishes at a disadvantage to native ones is necessary for suppressing invasions, but the knowledge is scarce, particularly when abiotic changes are fast. In this study, we increased this knowledge by an analysis of the biomass of most harmful Prussian carp *Carassius gibelio* in a river reviving from biological degradation. The species' invasion followed by the invasion's reversal occurred over only two decades and were documented by frequent monitoring of fish biomass and water quality. An initial moderate improvement in water quality was an environmental filter that enabled Prussian carp’s invasion but prevented the expansion of other species. A later substantial improvement stimulated native species’ colonization of the river, and made one rheophil, ide *Leuciscus idus*, a significant Prussian carp’s replacer. The redundancy analysis (RDA) of the dependence of changes in the biomass of fish species on water quality factors indicated that Prussian carp and ide responded in a significantly opposite way to changes in water quality in the river over the study period. However, the dependence of Prussian carp biomass on ide biomass, as indicated by regression analysis and analysis of species traits, suggests that the ecomorphological similarity of both species might have produced interference competition that contributed to Prussian carp’s decline.

## Introduction

Non-native species are a major driver of the biotic homogenization and loss of biodiversity of freshwater ecosystems^1–3^. Among non-native freshwater fish species, Prussian carp *Carassius* (*auratus*) *gibelio* (Bloch 1782)^4^ is probably the second most harmful such driver beside common carp^5^ in the temperate climatic zone^6–9^. The cause of Prussian carp’s harmfulness is their ability to establish in and quickly invade freshwater ecosystems of any scale, particularly in Eurasia^10–12^. This ability has long produced a need for inventing methods of suppressing Prussian carp, which should be easy in view of an extensive knowledge of Prussian carp’s traits^13–17^. Yet, the whole knowledge is of traits that put Prussian carp at an advantage to other fish species. Meanwhile, the knowledge of traits that put Prussian carp at a disadvantage to other fish species is necessary for developing suppressing methods and such knowledge is almost absent.

There are two exceptions to this absence of knowledge about factors that put Prussian carp at a disadvantage to other species. One of them is the vulnerability of Prussian carp to herpesviral hematopoietic necrosis virus (cyprinid herpesvirus 2, CyHV-2)^18–20^. The other is decrease in Prussian carp abundance and invasiveness while desiccation events are avoided^21^. The virus is a biotic while the avoidance of desiccation events an abiotic driver of Prussian carp’s extirpation. Yet, it is noticeable that both were identified owing to effects observed in natural and/or cultured environments in the course of observational studies.

**Figure 1:**
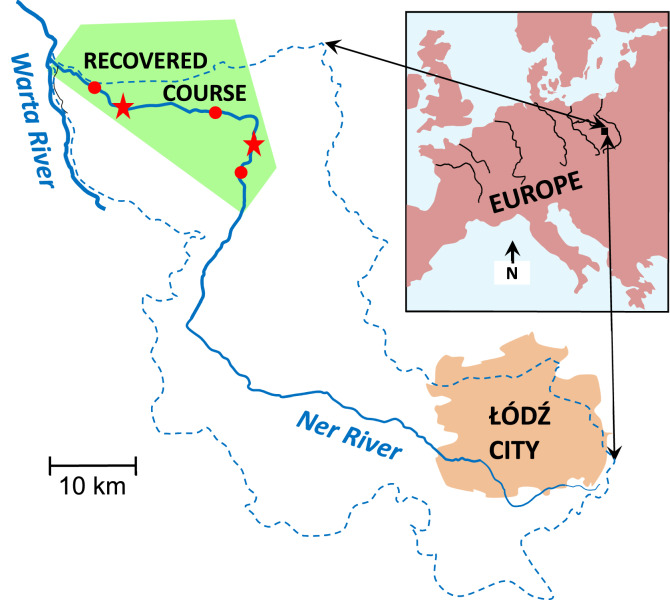
Ner River, Poland, and its location in Europe. Red dots situated along the Ner are fish sampling sites in the recovered course. The left asterisk is the Dąbie water quality monitoring station, the right asterisk is the Podłęże water quality monitoring station. Dashed blue line is the Ner catchment limit. River degradation gradient extended from the Łódź City (worst condition) to the downstream end of the recovered course (best condition). Map of this figure was drawn by the present Authors using Microsoft PowerPoint v. 2010 software on the basis of other maps of the study area.

The importance of observational studies for detecting drivers that are harmful to Prussian carp is confirmed by the present study. The study describes another case of Prussian carp’s extirpation and a successful search for the drivers of the extirpation. The case took place in a river reviving from long environmental degradation^22,23^. The degradation occurred in the middle (about 60 km long) and lower (about 40 km long) courses of the lowland Ner River, Poland (Fig. 1). The degradation lasted for almost the whole twentieth century, during which the courses were almost fishless. Pollution abatement and improvement in oxygen conditions began there in the 1990s. The abatement and improvement resulted in gradual decrease in environmental stress put on fish that continues today. As a result, Ner fish fauna has much recovered, which was indicated by seven sampling surveys made along the whole river in 2000–2012 (see Penczak et al.^24^, for details), in the course of which fish species’ incidence, abundance and biomass were determined.

The structure of the fish recovery evolved both temporally and spatially as regards all fish species. By 2000 Prussian carp had dominated the lower course (with the native weatherfish *Misgurnus fossilis*) and the middle course. By 2012, these two species had been almost completely replaced in the lower course by numerous native species, ide (*Leuciscus idus*) having become the decisive dominant. It is presumed that the direct driver of the fish recovery in the Ner was improvement in water quality of the river, which acted as an environmental filter^24–26^. The initial phase of the improvement (up to 2000) made both the middle and lower courses good enough mostly for Prussian carp (and the lower one also for weatherfish). The later phase (2002 through 2012) made the lower and middle courses suitable also for other fish species, although for a high number of these species only the lower course. However, it was also considered that the restored native fish species might have been biotic drivers contributing to the extirpation of Prussian carp^27,28^.

The Ner fish recovery is connected to two major problems of ecology: the relation between native and non-native species and environmental influence over biodiversity. Yet, the recovery was untypical in many respects, such as the appearance and disappearance of a single and very specific non-native species, and a very fast and very deep environmental change, which presumably was the cause of the very rapid recovery of numerous native species. The specific questions that we asked and answered in this study were: (1) Which water quality variables explained the observed changes in given native fish species biomass in the lower course of the Ner (i.e. the recovery)?, (2) Which water quality variables explained the decline in the non-native Prussian carp in the lower course?, (3) Did biotic drivers in the form of native fish species also contribute to the Prussian carp extirpation?

## Material and methods

### Environmental variables, study sites and fish data

The Ner River is 124 km long and flows from the Łódź City to the Warta River, Poland. The river’s discharge is 11 m^3^ s^−1^ at the outlet. In the upper course, the discharge is only 1 m^3^ s^−1^ but it is additionally increased by approximately 3–4 m^3^ s^−1^ of water disposed to the downstream end of the upper course from the Łódź City Sewage Treatment Plant (see “Discussion”). In Table 1, the values of 14 most important physical and chemical water quality variables in the recovered Ner course, which is the lower course of the river mentioned above, are presented for several years in the sampling period. The values were collected at the Dąbie and Podłęże water quality monitoring stations of the Poznań and Łódź Voivodeship Inspectorates of Environmental Protection, located at 12.8 and 34.2 km of the Ner (measured from the river’s outlet), respectively. The value of every variable in every year included in Table 1 is the mean obtained from samples collected once a month in the summer months (May, June, July, August) of each year. The dates of sampling differed from month to month and from year to year.

**Table 1:** Mean values of water quality variables from samples obtained in summer months (May–August) in the recovered course of the Ner River, Poland. Abbreviations of variable names that are used in the RDA figure are given (if different from those used here) in brackets.

Water quality variable	Unit	Year						
		2000	2002	2004	2005	2008	2009	2011
Suspended solids	mg/l	19.00	18.67	10.48	9.95	7.65	8.30	11.30
pH		7.30	7.58	7.53	7.73	7.73	7.78	7.95
Dissolved oxygen (DO)	mg O_2_/l	4.35	6.30	7.50	8.50	8.65	8.40	9.20
BOD_5_	mg O_2_/l	9.88	4.18	4.10	4.63	2.93	3.10	2.90
Ammonia nitrogen	mg N/l	4.06	0.46	2.62	2.50	0.16	0.23	0.09
Nitrates (NO_3_)	mg N/l	1.25	2.61	4.47	3.38	2.70	3.68	3.15
Total nitrogen	mg N/l	9.41	6.70	10.68	7.65	6.17	6.50	5.68
Total phosphorus (T. Phosph.)	mg P/l	1.78	1.00	0.86	0.62	0.51	0.45	0.31
Conductivity (Conduct.)	μS/cm	726.75	778.00	961.50	771.00	1032.75	783.50	706.25
Alkalinity	mg CaCO_3_/l	257.00	293.30	327.33	239.75	224.13	235.50	237.00
Sulphates	mg SO_4_/l	61.00	60.00	77.38	72.25	89.25	77.40	76.00
Chlorides	mg Cl/l	74.25	83.00	98.43	74.88	126.43	94.73	76.25
Calcium (Ca)	mg Ca/l	69.63	98.60	107.63	82.06	72.75	80.00	79.25
Magnesium	mg Mg/l	8.13	11.50	10.96	9.04	10.38	8.75	9.40

Fish species biomass data for the whole Ner were obtained by CPUE single run electrofishing in 10–14 sites at 1–3 years intervals. Three of the sites were located in the recovered course and they supplied all samples that are considered in the present study. Direct electric current was used, which only stuns fish and does not kill them. After identification and measuring fish length and weight all fish individuals were released to river water. The applied fish sampling protocols were congruent with the institutional guidelines of the University of Łódź, and the sampling team possessed valid personal licenses to apply such protocols. The protocols were approved either by the Rector of the University of Lodz (Łódź) or the Dean of the Faculty of Biology and Environmental Protection of the University of Lodz (Łódź) on the basis of “Ustawa o Ochronie Zwierząt” = “Law on the Protection of Animals”, published in Dziennik Ustaw No. 111, Item 724, of 21 August 1997 (in Polish), and “Zasady Wykonywania Doświadczeń na Zwierzętach i Tryb Uzyskiwania Zezwoleń na Prowadzenie Eksperymentów” = “Rules of Carrying out  Experiments on Animals and Procedures for Obtaining Permits to Carry on such Experiments”, published in Zarządzenie Rektora No. 35 of 5 February 2001 (in Polish), and applied in accordance with the Polish and European Union guidelines and regulations that were then valid, i.e. Dziennik Ustaw No. 6 Item 17 of 4 February 1980 (in Polish), EN 25667-1:1993 (ISO 5667-1:1980), IEC 60335-2-86, Council Directive 92/43/EEC of 21 May 1992 and CEN EN 14011:2003. See Penczak et al.^24^ for the location of all study sites and details of fish sampling in the Ner. Here, in contrast to the Penczak et al.^24^ study, fish biomass is analysed at the species level, the biomass of each species constituting the total from samples obtained in the three sites of the section (Fig. 1) in a given (one of seven) sampling survey.

### Statistical analyses

The first two questions of the study that are posed in the Introduction might be answered by multivariate statistical analysis applied to test the dependence of the biomass values of fish species, which are specified in Table S1, on the values of water quality variables, which are presented in Table 1. Before the analysis outliers were removed from the variables of the table. All variables were then correlated to determine collinearity (Table 2), and further analysis was carried out using only those variables that were uncorrelated. Neither the values of water quality variables nor of biomass variables were transformed. Initially, Detrended Correspondence Analysis (DCA) was performed to determine the length of gradients. The length was 2.712, which indicated a linear response of the data and allowed us to apply one of linear analyses. The selected linear analysis was Redundancy Analysis (RDA). A forward selection was carried out with Monte Carlo permutation tests to estimate the contribution of each water quality variable to the explained variance of the response variables, i.e. fish species biomass. Both DCA and RDA analyses were carried out with CANOCO 4.5 software^29^.

**Table 2:** Pearson correlation coefficients (half of the table above the diagonal) between the values of environmental variables measured in the recovered course of the Ner River, Poland, in the fish sampling period, and significance values of these correlation coefficients (half of the table below the diagonal). The number of values was 7 in all variables, degrees of freedom were 5 in all tests. Statistically significant values are in italic font. Abbreviations of environmental variables’ names: *DO* dissolved oxygen, *BOD*_*5*_ biological oxygen demand, *NO*_*3*_ nitrates, *T. phosph.* total phosphorus, *Conduct.* conductivity, *Ca* calcium, *Discharge* discharge, *S.* *solids* suspended solids, *pH* pH, *A. nitrogen* ammonia nitrogen, *Nitrites* nitrites, *T. nitrogen* total nitrogen, *Phosph.* phosphates, *Alkalinity* alkalinity, *Sulphates* sulphates.

Water quality variable	DO	BOD_5_	NO_3_	T. phosph.	Conduct.	Ca	Discharge	S. solids	pH	A. nitrogen	Nitrites	T. nitrogen	Phosph.	Alkalinity	Sulphates
DO		− *0.8867*	0.6684	− *0.9825*	0.2467	0.0076	− 0.4791	− *0.8684*	*0.9285*	− 0.6685	− *0.8628*	− 0.5537	− *0.9673*	− 0.3985	*0.7709*
BOD_5_	*0.008*		− 0.7102	*0.9414*	− 0.3572	− 0.2986	0.2224	0.7059	− *0.8377*	*0.8483*	*0.8221*	0.5754	*0.9293*	0.1289	− 0.6571
NO_3_	0.101	0.074		− 0.6644	0.3497	0.6377	− 0.3786	− 0.6768	0.4712	− 0.2800	− 0.3956	0.1028	− 0.6425	0.3271	0.4983
T. phosph.	*0.000*	*0.002*	0.104		− 0.2059	− 0.0587	0.3469	*0.8114*	− *0.9509*	*0.7803*	*0.9174*	0.6358	*0.9941*	0.3704	− 0.7126
Conduct.	0.594	0.432	0.442	0.658		0.2383	− 0.5678	− 0.5033	− 0.0540	− 0.1368	0.1125	0.1874	− 0.1270	0.1893	0.6939
Ca	0.987	0.515	0.123	0.901	0.607		0.1740	0.0805	− 0.1123	− 0.0031	0.1611	0.4131	− 0.0449	*0.8895*	− 0.1512
Discharge	0.277	0.632	0.402	0.446	0.184	0.709		0.7229	− 0.2383	− 0.0642	0.0972	− 0.1922	0.3136	0.1806	− *0.8155*
S. solids	*0.011*	0.076	0.095	*0.027*	0.250	0.864	0.066		− 0.6658	0.4183	0.6057	0.2603	*0.7712*	0.3806	− *0.9213*
pH	*0.003*	*0.019*	0.286	*0.001*	0.908	0.811	0.607	0.103		− *0.7847*	− *0.9751*	− *0.7644*	− *0.9690*	− 0.5111	0.5671
A. nitrogen	0.101	*0.016*	0.543	*0.038*	0.770	0.995	0.891	0.350	*0.037*		*0.8555*	*0.8521*	*0.8002*	0.3459	− 0.4563
Nitrites	*0.012*	*0.023*	0.380	*0.004*	0.810	0.730	0.836	0.149	*0.000*	*0.014*		*0.8468*	*0.9429*	0.5594	− 0.4990
T. nitrogen	0.197	0.177	0.826	0.125	0.687	0.357	0.680	0.573	*0.045*	*0.015*	*0.016*		0.6619	0.7035	− 0.2678
Phosph.	*0.000*	*0.002*	0.120	*0.000*	0.786	0.924	0.493	*0.042*	*0.000*	*0.031*	*0.001*	0.105		0.3764	− 0.6700
Alkalinity	0.376	0.783	0.474	0.413	0.684	*0.007*	0.698	0.400	0.241	0.447	0.192	0.078	0.405		− 0.3674
Sulphates	*0.042*	0.109	0.255	0.072	0.084	0.746	*0.025*	*0.003*	0.184	0.303	0.254	0.561	0.100	0.418	

The results of RDA analysis are presented in the text, tables and in a plot. Green vectors of the plot indicate fish biomass values, red vectors water quality variables. Axis x of the plot represents Axis 1 and axis y represents Axis 2 of the RDA analysis. A small angle between vectors of the plot indicates a strong positive correlation, an angle close to 90° lack of correlation, and an angle close to 180° a strong negative correlation. The results of RDA analysis were also assessed by the application of Generalized Linear Models (GLM) with the gamma distribution and log-link function. The GLM models were applied to relate the biomass of Prussian carp and ide to RDA Axis 1 and to water quality variables.

Regression analysis was applied to investigate the relation between the biomass of specific pairs of fish species, in particular the dependence of Prussian carp biomass on the biomass of each of the other species. For this analysis all the biomass data were Hellinger transformed. This transformation consists of dividing each element, i.e. a given biomass value, by the raw sum, i.e. the total biomass in a given sampling survey, and of square rooting of each element. This transformation made the surveys suitable for a number of statistical analyses and reduced the impact of high biomass species. Hellinger transformation avoids the Orloći paradox^30^, for example.

Model II regression was applied to search for the biotic drivers because both the response and explanatory variables of the models were random, i.e. not controlled by the researcher^31,32^. OLS regression lines always underestimate slopes when both variables are random^33^. However, OLS models of the same data set are presented (Table S2) to indicate to what extent results of model I regression may differ from model II regression. There are several model II regression methods and usually one of them is (most) correct in a given situation. In our case, the major (main, principal) axis method, MA^31,33,34^, was suitable. Firstly, because it might be presumed that error variance of any random variable (i.e. a species’ biomass) of a given pair was similar (owing to same river, same sites, same terms of sampling, same sampling technique and equipment, and same reaction of species to electric current). Secondly, because both variables of each pair used in a regression were approximately bivariate normal.

The significance of an MA model depends on the significance of the slope of the major axis, which is determined by permutation^32,33^, and it was also used as the first and major method of comparing MA models. The reference value used under permutation for assessing the significance of MA slope is the slope value itself when it is smaller than 1 and larger than − 1, and its reciprocal otherwise^34^. One-tailed probabilities are computed in the direction of the sign of the coefficient.

The significance values of MA regression models were corrected for multiple hypotheses using Holm^35^ adjustment, because the hypotheses were obviously non-independent as all the fish species inhabited the same environment and thus interacted. Holm^35^ adjustment accounts for such non-independence, but requires no assumptions about its structure, which was unknown to the authors.

Besides, the mean of a bivariate normal distribution (where major and minor axes, and confidence limit lines cross) may be tested for the alpha (95%) confidence region around the sample mean. The shape and size of this region is dependent on the eigenvalues of the variance–covariance matrix of both variables. It indicates the intensity of relation between the variables, and was the second method of comparing MA models. This region is an ellipse, whose elongated form (indicating a strong dependence of one variable on the other) occurs when a great value of one eigenvalue, which represents the first principal component, occurs with a small magnitude of the other, which represents the second (minor) component. If eigenvalues are similar the ellipse is close to a circle^31^.

The same major axis methodology was also applied to untransformed biomass data. In this way the dependence of the biomass of Prussian carp on the biomass of other fish species was additionally tested.

## Results

**Figure 2:**
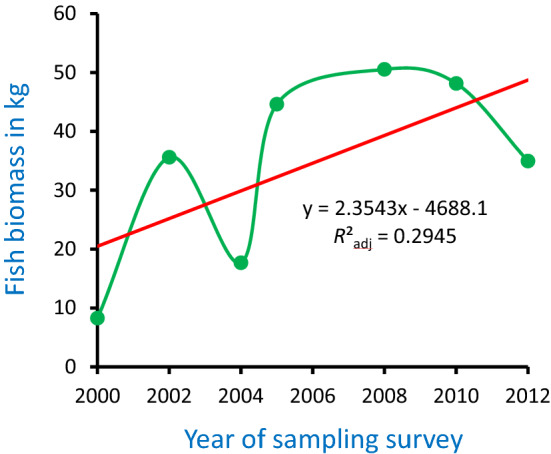
Total biomass of all fish species in the recovered course of the Ner River, Poland, in subsequent sampling surveys over the period of 2000–2012.

The recovered course of the Ner supplied fish samples that weighed 240 kg over the study period. Complete untransformed biomass data obtained on each sampling occasion for all fish species for which regression models could be constructed are presented in Table S1. (See Penczak et al.^24^, for the complete fish species list of the river, and Supplementary data (mmc12—Table S2) of Penczak et al.^24^ for fish species richness, biomass and abundance in given sections in given sampling surveys). In the recovered course, the number of all species increased from six to 22, of fluvial specialist species (rheophils) from zero to six, and the total fish species biomass from 8 to 34 kg between the first and last surveys (Table 3, Fig. 2).

**Table 3:** Fish biomass in the recovered course of the Ner River, Poland, at the beginning and end of the fish sampling period (percentages were obtained from untransformed biomass values, see Table S1).

Fish species	Percentage of the biomass of all fish species in the recovered course	
	Year 2000	Year 2012
Fluvial specialist species (rheophils): *Lota lota—*burbot *Leuciscus cephalus—*chub *Leuciscus leuciscus*—dace *Leuciscus idus—*ide *Barbatula barbatula—*stone loach *Gobio gobio—*gudgeon	0.00	37.03
*Carassius gibelio—*Prussian carp	59.27	0.04
*Misgurnus fossilis—*weatherfish	40.32	0.01
*Leuciscus idu*s—ide	0.00	30.58
*Rutilus rutilus—*roach	0.30	21.86
*Perca fluviatilis—*perch	0.05	17.24

**Figure 3:**
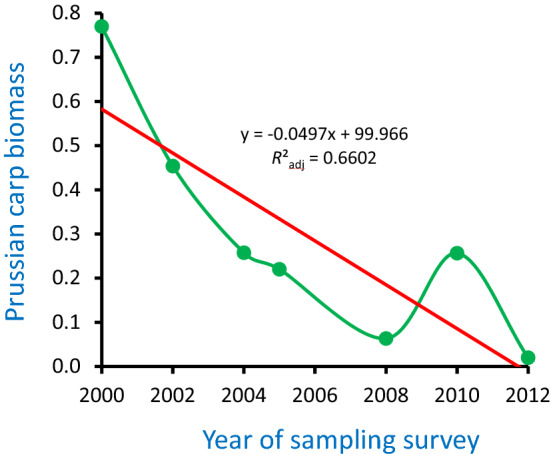
Prussian carp biomass decline in the recovered course of the Ner River, Poland, over the fish sampling period of 2000–2012. Green dots are Hellinger transformed values of *Carassius gibelio*—Prussian carp biomass in each of the sampling surveys. Red line is linear regression of the values.

Changes in the total raw fish biomass in the recovered course over the sampling period were not significant (biomass = 2.3543x - 4688.1, *F*_1,5_ = 3.50, *P*-value = 0.120, *R*^2^_adj_ = 0.2945) (Fig. 2) when they were assessed by an OLS model (model I regression). Changes in Prussian carp biomass in the recovered course over the period were statistically significant for Hellinger transformed data (biomass = -0.0497x + 99.966, *F*_1,5_ = 12.66, *P*-value = 0.016, *R*^2^_adj_ = 0.6602) (Fig. 3) when they were also modelled by OLS. The slope of the regression model was negative. Fish community structure in the recovered course was greatly altered over the period: the biomass of rheophilic species increased from zero to one-third of the total assemblage biomass, while two initial dominant fishes (Prussian carp and weatherfish) were replaced by three new colonists-dominants (ide, roach and perch) (Table 3).

**Figure 4:**
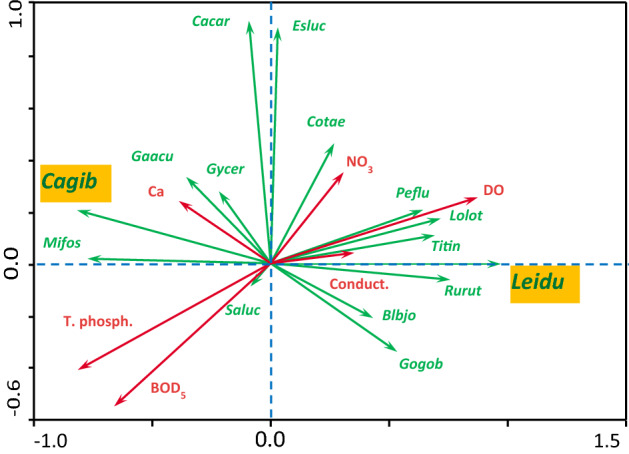
RDA biplot of water quality variables presented in Table 1 (after deleting collinear variables) and fish species’ biomass values obtained from samples collected in the recovered course of the Ner River, Poland, in 2000–2012. Abbreviations of water quality variables: *DO* dissolved oxygen, *BOD*_*5*_ biological oxygen demand, *NO*_*3*_ nitrates; *T. Phosph.* total phosphorus, *Conduct.* conductivity, *Ca *calcium. Abbreviations of fish species’ names: *Blbjo*—*Blicca bjoerkna* (silver bream); *Cacar*—*Carassius carassius* (crucian carp); *Cagib*—*Carassius gibelio* (Prussian carp); *Cotae*—*Cobitis taenia* (spined loach); *Esluc*—*Esox lucius* (pike); *Gaacu*—*Gasterosteus aculeatus* (stickleback); *Gogob*—*Gobio gobio* (gudgeon); *Gycer*—*Gymnocephalus cernua *(ruffe); *Leidu*—*Leuciscus idus* (ide); *Lolot*—*Lota lota* (burbot); *Mifos*—*Misgurnus fossilis* (weatherfish); *Peflu*—*Perca fluviatilis* (perch); *Rurut*—*Rutilus rutilus* (roach); *Saluc*—*Sander lucioperca* (pikeperch); *Titin*—*Tinca tinca* (tench).

Changes in the values of 14 important physical and chemical water quality variables in the recovered course of the Ner indicate that the values of such variables as pH, dissolved oxygen, or sulphates much increased, while those of suspended solids, BOD_5_, ammonia nitrogen, total nitrogen and total phosphorus much decreased (Table 1). The results of the RDA analysis (Table 4; Fig. 4) show that four distinguished canonical axes explained 86.2% of total variation (sum of all canonical eigenvalues) in the fish biomass and water quality variables obtained in the recovered course.

**Table 4:** Results of RDA analysis of the relations between fish biomass and water quality variables. Abbreviations of fish species names that are used in the RDA figure are given in brackets. Statistically significant variables are in bold font. The respective values of significant variables are in italic font. (A)—Variability of fish species biomass (in shares of unity) explained by given canonical axis. (B)—Correlations between the biomass of given fish species and the canonical axes representing species. (C)—Correlations between water quality variables and canonical axes representing water quality variables.

	Axis 1	Axis 2
**(A)**		
Eigenvalue	0.479	0.243

Fish species	Species Axis 1	Species Axis 2
**(B)**		
*Blicca bjoerkna*—silver bream (*Blbjo*)	0.432	− 0.209
***Carassius carassius***—**crucian carp** (***Cacar***)	− 0.092	*0.930*
***Carassius gibelio*—Prussian carp** (***Cagib***)	− *0.817*	0.204
*Cobitis taenia*—*spined loach* (*Cotae*)	0.268	0.459
***Esox lucius***—**pike** (***Esluc***)	0.030	*0.904*
*Gasterosteus aculeatus*—stickleback (*Gaacu*)	− 0.356	0.331
*Gogio gobio*—gudgeon (*Gogob*)	0.533	− 0.339
*Gymnocephalus cernua*—ruffe (*Gycer*)	− 0.218	0.276
***Leuciscus idus***—**ide** (***Leidu***)	*0.973*	0.001
*Lota lota*—burbot (*Lolot*)	0.718	0.172
*Misgurnus fossilis*—weatherfish (*Mifos*)	− 0.772	0.018
*Perca fluviatilis*—perch (*Peflu*)	0.643	0.205
*Rutilus rutilus*—roach (*Rurut*)	0.757	− 0.063
*Sander lucioperca*—pikeperch (*Saluc*)	− 0.085	− 0.088
*Tinca tinca*—tench (*Titin*)	0.692	0.108

Water quality variable	Water quality Axis 1	Water quality Axis 2
**(C)**		
**Dissolved oxygen (DO)**	*0.874*	0.253
**Biological oxygen demand (BOD**_**5**_**)**	− *0.661*	− 0.548
Nitrates (NO_3_)	0.307	0.349
**Total phosphorus (T. phosph.)**	− *0.813*	− 0.409
Conductivity (Conduct.)	0.352	0.041
Calcium (Ca)	− 0.390	0.231

RDA Axis 1 and 2 were much more important for explaining variation in the species and water quality data (72.2%) than RDA Axis 3 and 4 (12.7%). There was a strong correlation (0.988) between RDA Species Axis 1 and RDA Water Quality Axis 1. RDA Axis 1 explained 47.9% (Table 4A) of species biomass variation and 55.6% of the relation between species biomass and water quality variables. RDA Species Axis 1 was significantly positively correlated only with the biomass of ide (0.973), and significantly negatively only with the biomass of Prussian carp (-0.817). RDA Species Axis 2 was significantly positively correlated only with the biomass of crucian carp (0.930) and pike (0.904) (Table 4B). The correlation between given water quality variables and RDA Water Quality axes was significantly positive only between RDA Water Quality Axis 1 and dissolved oxygen (0.874) and significantly negative only between RDA Water Axis 1 and total phosphorus (-0.813) and biological oxygen demand (-0.661) (Table 4C). The conditional effects resulting from the forward stepwise selection of water quality variables consisted in only dissolved oxygen being significantly important for species biomass (LambdaA = 0.39, *F* = 3.22, *P* = 0.028).

In the plot of Fig. 4, changes in the biomass of fish species are graphically related to changes in water quality variables as regards RDA Axes 1 (axis x) and RDA Axis 2 (axis y). The vectors of Prussian carp and ide, for example, are directed in opposite directions, which indicates that a decrease in Prussian carp biomass co-occurred with increase in ide biomass. Each of these changes was differently related to changes in water quality variables, dissolved oxygen content favouring high ide biomass and low Prussian carp biomass, for example.

**Figure 5:**
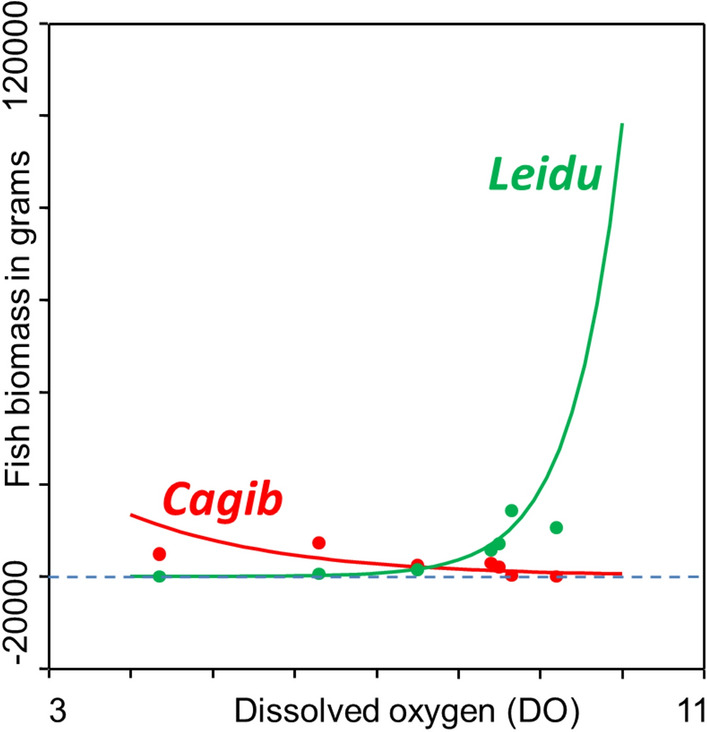
GLM model of the dependence of *Carassius gibelio—*Prussian carp (*Cagib*) and *Leuciscus idus*—ide (*Leidu*) biomass on dissolved oxygen in the recovered section of the Ner River, Poland in 2000–2012. Red and green dots are characteristic points of the GLM model. Dissolved oxygen (DO) was measured in mg O_2_/l.

GLM analysis indicated that only the biomass of ide was significantly (and positively) related to RDA Axis 1 (Table 5). Also, only the biomass of ide was significantly related to water quality variables: positively to dissolved oxygen content (Fig. 5) and negatively to calcium and total phosphorus contents (Table 5).

**Table 5:** GLM analysis of relations between RDA Axis 1 and the biomass of *Carassius gibelio*—Prussian carp and *Leuciscus idus—*ide and between water quality variables and the biomass of *Carassius gibelio* or *Leuciscus idus.* Symbols of variables that were used in the RDA figure are in brackets. The species whose biomass significantly correlated with a given water quality variable are in bold font, and significant *P*-values are in italic font.

RDA axis	Species	Intercept—a	SE of a	Slope—b	SE of b	*F*	*P*-value
Axis 1	*Carassius gibelio* (*Cagib*)	7.575	0.354	− 0.849	0.350	4.98	0.076
	***Leiciscus idus* (*Leidu*)**	7.858	0.257	1.634	0.254	23.67	*0.005*

Water quality variable	Species	Intercept—a	SE of a	Slope—b	SE of b	*F*	*P*-value
Dissolved oxygen (DO)	*Carassius gibelio* (*Cagib*)	11.627	1.850	− 0.530	0.240	3.77	0.110
	***Leiciscus idus* (*Leidu*)**	− 5.033	1.865	1.653	0.242	16.34	*0.010*
Total phosphorus (T. phosph.)	*Carassius gibelio* (*Cagib*)	6.504	0.805	1.520	0.883	2.26	0.193
	***Leiciscus idus* (*Leidu*)**	13.74	0.795	− 8.360	0.872	18.12	*0.008*
Calcium (Ca)	*Carassius gibelio* (*Cagib*)	6.803	2.676	0.013	0.031	0.169	0.302
	***Leiciscus idus* (*Leidu*)**	14.133	1.685	− 0.068	0.018	7.12	*0.044*

**Figure 6:**
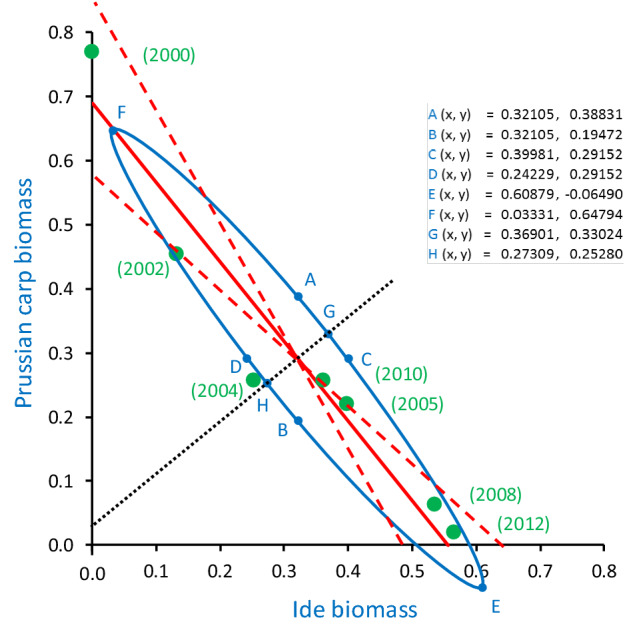
*Carassius gibelio—*Prussian carp biomass as a function of *Leuciscus idus—*ide biomass (both Hellinger transformed) in the recovered course of the Ner River, Poland, in subsequent sampling surveys over the period of 2000–2012. Green dots are biomass values in given surveys, while green numbers in brackets are years of the surveys. Red solid line is the major axis (of the bivariate distribution and of the ellipse). Red dashed lines are 95% confidence limits of the major axis. Black dotted line is the minor axis. The blue ellipse is 95% confidence region of the mean of both variables. Blue dots (marked by blue letters, ‘A…H’), are characteristic points of the ellipse. The coordinates of the points (e.g. ‘A (x, y) =’) are also indicated, x being the abscissa, y the ordinate axis location.

Model II regression applied to Hellingertransformed data indicated that the biomass of Prussian carp depended on the biomass of seven fish species, but only ide remained significant after the Holm^35^ adjustment (major axis slope = −1.239x, *P*-value = 0.0028) (Table 6), which considered 14 species for which regression models could be constructed. This dependence is also indicated in Fig. 6 by a narrow angle between the 95% confidence limit lines of the MA regression line (i.e. its principal axis) of the two species, and a very elongated ellipse of the 95% confidence region of the bivariate mean. The MA regression models of the dependence of weatherfish biomass on other fish species biomass are presented in Table S3, and the plot of the dependence of Prussian carp biomass on *Rutilus rutilus—﻿*roach biomass is shown in Figure S1.

**Table 6:** Major Axis (MA) model II regressions used to assess the dependence of the biomass of *Carassius gibelio*—Prussian carp on the biomass of other fish species that were sampled in the recovered course of the Ner River, Poland, over the period of 2000–2012. Hellingertransformed data were used. Number of cases (i.e. of sampling surveys) was 7 in all models; na—data not available (which results from small difference between λ1 and λ2, and also an H value higher than 1). ^‡^Monotonicity adjustment.

Fish species (scientific names—common names)	Eigenvalues		H statistic	Minor axis		Major axis		Angle (degrees)	*P*-value (1-tailed)	Holm adjusted *P*-value	95% Confidence limits of intercept of major axis		95% Confidence limits of slope of major axis	
	λ1	λ2		Intercept	Slope	Intercept	Slope				2.5%—Intercept	97.5%—Intercept	2.5%— Slope	97.5%— Slope
*Leuciscus idus*—ide	0.106	0.002	0.025	0.032	0.807	0.689	− 1.239	− 51.086	0.0002	0.0028	0.851	0.581	− 1.743	− 0.901
*Rutilus rutilus*—roach	0.090	0.006	0.098	0.038	0.656	0.880	− 1.525	− 56.745	0.0123	0.1599	1.727	0.599	− 3.719	− 0.797
*Misgurnus fossilis*—weatherfish	0.120	0.005	0.063	0.450	− 0.964	0.120	1.038	46.058	0.0171	0.2052	0.191	− 0.002	0.612	1.778
*Tinca tinca*—tench	0.080	0.010	0.225	0.187	0.529	0.665	− 1.890	− 62.116	0.0262	0.2882	− 24.406	0.424	124.810	− 0.669
*Alburnus alburnus*—bleak	0.066	0.001	0.023	0.286	0.114	0.690	− 8.761	− 83.488	0.0355	0.3550	− 0.862	0.458	25.383	− 3.660
*Blicca bjoerkna*—silver bream	0.069	0.005	0.100	0.262	0.250	0.767	− 3.999	− 75.962	0.0365	0.3550^‡^	− 1.263	0.478	13.090	− 1.573
*Gobio gobio*—gudgeon	0.070	0.006	0.133	0.254	0.295	0.716	− 3.386	− 73.548	0.0452	0.3616	− 1.154	0.453	11.531	− 1.286
*Perca fluviatilis*—perch	0.079	0.007	0.142	0.129	0.492	0.964	− 2.034	− 63.825	0.0738	0.5166	0.142	0.586	4.940	− 0.892
*Lota lota*—burbot	0.068	0.004	0.091	0.266	0.227	0.784	− 4.408	− 77.219	0.0794	0.5166^‡^	− 1.042	0.482	11.951	− 1.708
*Cobitis taenia*—spined loach	0.065	0.000	0.001	0.291	0.022	1.192	− 45.057	− 88.729	0.1355	0.6775	− 1.195	0.637	74.352	− 17.273
*Esox lucius*—pike	0.080	0.032	1.515	0.020	0.681	0.879	− 1.469	− 55.760	0.1944	0.7776	na	na	na	na
*Gasterosteus aculeatus*—stickleback	0.065	0.000	0.004	0.292	− 0.019	− 0.492	51.859	88.895	0.2113	0.7776^‡^	0.100	0.669	12.701	− 24.979
*Sander lucioperca*—pikeperch	0.065	0.004	0.080	0.288	0.082	0.788	− 12.263	− 85.338	0.2238	0.7776^‡^	0.097	0.397	4.804	− 2.594
*Gymnocephalus cernua*—ruffe	0.065	0.000	0.008	0.291	0.024	1.626	− 41.996	− 88.636	0.2544	0.7776^‡^	− 0.199	0.573	15.439	− 8.868

When model II regression was applied to untransformed fish biomass data only the dependence of Prussian carp biomass on ide biomass turned out significant (slope of the major axis = − 0.393x, *P*-value = 0.0226) (Table 7). However, when the model was Holm-adjusted (also using 14 fish species for which fish biomass regression models could be constructed), the *P*-value became 0.3166, and thus the model became insignificant.

**Table 7:** Major Axis (MA) model II regression used to assess the dependence of the biomass of *Carassius gibelio*—Prussian carp on the biomass of *Leuciscus idus*—ide that were sampled in the recovered course of the Ner River, Poland, over the period of 2000–2012. Untransformed biomass data (in grams) were used. Number of cases (i.e. of sampling surveys) was 7.

Fish species (scientific name—common name)	Eigenvalues		H statistic	Minor axis		Major axis		Angle (degrees)	*P*-value (1-tailed)	Holm adjusted *P*-value	95% Confidence limits of intercept of major axis		95% Confidence limits of slope of major axis	
	λ1	λ2		Intercept	Slope	Intercept	Slope				2.5%—Intercept	97.5%—Intercept	2.5% —Slope	97.5%—Slope
*Leuciscus idus*—ide	34,452,977	2,836,864	0.129	− 11,741.1	2.544	4884.863	− 0.393	− 21.458	0.0226	0.3166	7851.489	2698.484	− 0.917	− 0.007

In short, the obtained results answer the three questions put forward in the Introduction. By using RDA and GLM analyses changes in fish species biomass were related to changes in specific water quality variables, which were identified. Dissolved oxygen, total phosphorus, BOD_5_, and perhaps calcium, were these variables that explained most of the changes in fish biomass, including the decrease in Prussian carp biomass and increase in the biomass of numerous native species, particularly ide. Besides, the strong dependence of Prussian carp biomass on ide biomass, as indicated by model II regression, suggested that ide may have been a biotic driver essentially contributing to Prussian carp decline.

## Discussion

The Ner River has been for decades the major route of disposing sewage and storm water from the Łódź City, a million people municipality located on the upper course of the river^24^. The improvement in water quality, and resulting fish recovery in the Ner, which are described in this study, was a consequence of two major processes that began in the early 1990s. Both these processes were management measures undertaken as part of the preparation for Poland’s accession to the European Community (now European Union), which took place in 2004. One of the processes was the liquidation of textile industry in the Łódź City, once one of the greatest textile production centers in the world^24^. The other of the processes was the modernization of agriculture and construction of numerous sewage purification stations in the Ner catchment, which took place over the 1990s and 2000s. The most important of the stations was the huge Łódź City Sewage Treatment Plant (STP), whose first part was launched in 1994. By 1995 all sewage disposed to the Ner (which was 3–4 m^3^/s) had been mechanically treated, by 1998 half of it had also been biologically treated, and since 2001 all of it has been biologically treated^24^, although the STP was further modernized in the whole 2000s. As a result of the above processes, oxygen content or transparency of the Ner River water much increased, while the load of nutrients or heavy metals much decreased in the study period.

There were three things that were essential for obtaining the significant fish analysis results that are presented above. One of them was frequent fish monitoring, which consisted of seven surveys. If the number of surveys over the period of 2000–2012 had been lower, say two or three, the intimate relation between Prussian carp and ide, for example, would not have been noticed, because no useful regression model could either be constructed or be significant. Such frequent monitoring as ours was exceptional in the early 2000s in Poland, and this is probably why the relation between the two fish species had not been detected before our study.

The frequent sampling was also little biased. Electrofishing, which was used in the surveys, might be reliably applied owing to several factors. Firstly, the recovered course was of slow water current, which resulted from a 17 m difference in elevation (and thus a 0.43‰ slope) between the upstream and downstream ends of the course. Such slow current made drifting of stunned fish too fast to be captured impossible. Secondly, turbidity which obstructs discernment of stunned fish, was low. Thirdly, conductivity was very stable, only once slightly exceeding 1000 μS/cm, and being 700–960 μS/cm on other sampling occasions (Table 1); such range of conductivity does not create technical or assessment problems of sampling efficiency or sampling selectivity^36^.

Finally, fish biomass data were standardized in a way that enabled constructing significant regression models. This occurred owing to the Hellinger transformation of data. Transformation of the data was necessary because of high variation in raw fish biomass between some of the sampling occasions.

### Prussian carp invasion, reversal of the invasion, recovery of the native fish species, and their drivers in the Ner

Results of the above analysis, in particular that of the RDA, indicate that the trait that enabled Prussian carp invasion of the recovered course in the phase of the initial environmental stress decrease was most probably the species’ ability to exist in worse oxygen conditions than other species. This is congruent with Prussian carp’s capacity for anaerobic metabolism, which is absent or weaker in other fish species^15,37^. Owing to this metabolism, Prussian carp can survive weeks of hypoxia, and even several hours of anoxia. Perhaps, other traits additionally enabling the invasion were Prussian carp’s tolerance of high phosphorus and nitrogen levels^16^, which were also noticed in the Ner in the late 1990s and early 2000s, and phenotypical plasticity of reproduction^12,38^.

The RDA results also indicate that additional factors favouring Prussian carp might have been high calcium and total phosphorus contents. In contrast, weatherfish were able to thrive and avoid competition with Prussian carp in the recovered course till 2000 owing to their ability to breath atmospheric air, detritus-oriented feeding tactics, and preference for vegetated zones of extremely shallow water depths^39,40^.

Yet, when the next phase of environmental stress decrease (over the course of the fish sampling period) made the recovered course of the Ner good enough to become colonisable by other fish species, the situation of Prussian carp changed dramatically. As the amount of dissolved oxygen further considerably increased in that period, the ability of anaerobic metabolism was no longer an asset, while the new colonizers became its competitors. Of these competitors ide may have been the most important species for Prussian carp decline (the causes of which are explained in the next subchapter). This is indicated by results of regression analysis presented in Tables 6 and 7 and Fig. 6 (see “Results”).

An open question is whether slower decrease in environmental stress than that presently observed in the recovered course would enable Prussian carp to develop defence mechanisms that would reduce their replacement by ide. This might be possible owing to Prussian carp’s phenotypical plasticity. This plasticity might produce modifications of the niche occupied by Prussian carp, and in this way lessened the interference competition between the two species. Unfortunately, there is no MA (or any other) model II regression that may be used with multiple predictors (and hence no such multispecies models are presented here), by analogy to multiple regression^31^. Multispecies model II regression might be useful because a probable long term interaction of Prussian carp with roach, for example, was observed by Paulovits et al*.*^41^, although it occurred in a shallow reservoir instead of a river.

### Why was ide the replacer of Prussian carp rather than other fishes?

The explanation why ide acted as the replacer of Prussian carp is difficult, but at least to some extent possible. Schiemer and Wieser^42^ defined food and feeding, ecomorphology, and energy assimilation and conversion as four groups of traits that decide about the success of given cyprinids, and used the traits to substantiate increasing roach dominance in Central European rivers. Although much less is known about these groups as regards ide (but see Rothla et al.^43^), yet ecomorphology seems to be most important also in its case. Large body depth of ide makes it similar to Prussian carp and thus its tough competitor. As the shape of ide is much less streamlined than that of most other large-bodied obligatorily riverine cyprinids, ide, like Prussian carp, avoids water current zone^44^ in order to reduce energy loss resulting from water resistance during movement. This increases the risk of occupying similar ecological niches by these two species. However, ide grow to bigger body sizes than Prussian carp, which gives the former a big advantage over the latter while searching for food (interference competition) and while avoiding predation.

Moreover, while Prussian carp is one of the most resistant fish species in general, ide belongs to the most resistant obligatory riverine (i.e. fluvial specialist) cyprinids, although its occurrence may sometimes even resemble that of limnophilic fishes^45,46^. The capacity of ide to be successful in more than averagely polluted river courses is manifest in the Warta, the parent river of the Ner. Przybylski^47^ and Kruk^46^, who distinguished contrasting reaches in the Warta, noticed a significantly higher biomass of ide in the middle, most polluted reach (to which the Ner empties), as early as in 1986–87 and 1996–1998, respectively. Ide usually dominated poor, several-species rich assemblages there. The situation was much similar in the Warta much later, in 2011–2012, when ide was significantly associated with the middle course, in which fish assemblages were in the poorest condition as compared to the upper and lower courses^48^.

Kruk^46^ attributes the high abundance of ide in the most polluted middle Warta River to weak competition from other rheophils, which were absent there because river degradation was too severe for them. In contrast, in the other sections of the Warta, ide were much less abundant owing to improved water quality and thus higher abundance of other rheophils, competitors of ide. If this presumption is correct, i.e. if the consequences of a spatial degradation gradient may become reflected in a temporal degradation gradient, then further decrease in environmental stress in forthcoming years may result in the replacement of ide by other rheophilic species in the Ner, too. This prognosis is supported by Eklöv et al.’s^45^ observation of ide decline coinciding with trout increase after a long-term improvement in water quality in streams of southern Sweden.

All fish species that colonized the recovered course of the Ner were species recorded for several dozen years in the Warta catchment^46,49–54^, and the fish species list of the catchment is about 20–40% longer than the list of species determined in the Ner. The list of the Warta is also similar to those of other nearby catchments of central Poland^55,56^. This indicates that all species that colonized the Ner in recent decades may have originated from the regional species pool^57,58^ rather than from stocking, aquaculture or unintentional introductions. Nevertheless, ide are frequently used in stocking, which increases their chance to become an instrument of controlling non-native fish species, while the present study contributes to the purposefull exploitation of the fish species. 

A quite different perspective of an invasion was presented by Bøhn et al.^59^. While monitoring the invasion of vendace (*Coregonus albula* L.) into upstream and downstream lakes 50 km apart located on the Norwegian sub-arctic Pasvik watercourse they observed great life history variability of the non-native fish entering a new environment. This consisted in decrease in the mean length in all age-classes, in fecundity, in the mean weight and size of individuals at first maturation, and increase in growth rate. Unfortunately, in the Ner we could only check the mean weight of individuals (results not shown): it varied in both Prussian carp and ide, but no clear decreasing or increasing trends were observed over the study period.

### Ide as the suppressor of Prussian carp, and other methods of extirpating the latter species

If the presumption that ide contributes as a biotic extirpator to Prussian carp decline is true then a comparison of ide with other suppression drivers is worth considering. One thing that may limit ide importance in other environments, for example, may be the above mentioned Prussian carp’s phenotypical plasticity: consequently, further research in this respect is necessary. Although the herpesviral hematopoietic necrosis virus (Cyprinid herpesvirus 2, CyHV-2) operates much faster than ide it cannot practically be used because it is uncontrollable in natural environments. This is the case because the virus, which is believed to have global occurrence, causes epizootics only when triggered by a specific range of water temperatures^60^, which of course can hardly be manipulated.

Besides, the virus suffers from the problem of selectivity. In the Czech Republic, the virus caused an epizootic that killed probably most individuals of numerous Prussian carp populations within weeks, but the fish were all triploid females^18^. It is not known why other ploidy forms^38^ were not affected, which is important because there is a natural tendency of invasive triploid female populations (with a few percent of males) to quickly transform themselves into diploid bisexual populations^12^. Moreover, first information about the virus indicated mass mortality of cultured goldfish [*Carassius auratus* (*auratus*)] in many countries, and it is not certain that it will not affect other fish species in the future^20^. Finally, the virus-assisted extirpation would be a very drastic form of animal control.

Reduction in frequency of desiccation events is an environmental measure of Prussian carp suppression that was discovered in Hungary^21^. It was observed there that in reservoirs, lakes and canals in which few or no desiccation events occurred, the relative abundance of Prussian carp constituted between one fifth and half of that recorded in fish ponds, for example, where desiccation was frequent. Moreover, the method is probably selective, affecting no other, native species. However, it cannot be applied to all freshwater bodies, for technical or financial reasons, and the elimination of Prussian carp is far from total. Interestingly, desiccation, and its relation to small water body sizes, was determined as one of factors favouring Prussian carp occurrence by Górski et al.^61^ in the Volga floodplain areas, where large water body size was also assessed as a factor favouring ide occurrence.

### Theoretical perspective

Generally, both the invasion by Prussian carp and its reversal comply with major theoretical predictions: the invasion with community ecology as a framework for biological invasions^62,63^ and the reversal with both the framework and the concept of biotic (ecological) resistance^27,28,64,65^. In the case of the invasion, because mostly the amount of resource (in this case: increase in dissolved oxygen, accompanied by decrease in BOD_5_, decrease in total phosphorus, etc.; in short—water quality) increased to a level that allowed the invader to exploit the environment, but was too low for other, native fishes, and thus Prussian carp (and weatherfish) colonized the river instead of the others. This also agrees with scenario 2 of the theoretical framework for invasions defined by Facon et al.^66^, in which environmental change is the main factor of invasion.

In the case of the reversal of the invasion, compliance with the theories occurs because the resource (mainly water quality) increased/improved high enough to be exploited by other, native species, and also because the native species became then competitors of the invader and thus biotic resistance drivers^23,28^. These drivers are defined in the biotic resistance hypothesis^64^, which describes the chances of an invasive species to be successful in a new environment. According to the hypothesis native-species-diverse environments are more resistant to invasive species than native-species-poor environments through a combination of predation, competition, parasitism, disease, and aggression. In this context, ide may resist Prussian carp, for example, owing to occupying similar spawning grounds as both species are open substratum spawners [ide being a phyto-lithophil (A.1.4), and Prussian carp a phytophil (A.1.5)]^67^. In the case of these two species, the resistance may be extended to ide predation on Prussian carp’ eggs, larvae or juveniles. Besides, ide grows to bigger body sizes than Prussian carp, which may result in aggressive behaviour in the form of scaring Prussian carp away from feeding grounds or hiding places.

In contrast, both the invasion and its reversal do not support the concept of invasional meltdown^68^, according to which in the initial phase an invasive species causes rapid changes in an ecosystem (by altering the trophic chain, for example), in this way paving the way for the invasion of subsequent non-native species^66^. In a next phase, when two or more alien species have invaded the ecosystem, synergistic interactions among them accelerate the invasion process^68^.

Yet, it is possible that the occurrence of biotic resistance rather than invasional meltdown has been an effect of insufficient biomass or abundance of other invasive species in the regional species pool^57,58^, of other aspects of the biotic context or small spatial and/or temporal scales of the processes^26^, or of environmental filters that might have prevented the invasion of other non-native species in the Ner^69^. Consequently, a number of quite different possible scenarios for the Ner are imaginable, for example no reversal of Prussian carp invasion if ide had not been abundant in the parent Warta River, or if species composition there had been quite different in other respects. This problem requires further research to reach reliable conclusions.

## Conclusions

The study indicated that fast water quality improvement produced fish species recovery in a previously degraded river. The recovery consisted of two phases, each one related to a different phase and degree of the improvement. The initial, moderate phase of the improvement enabled an invasion by non-native Prussian carp and native weatherfish, but served as an environmental filter that prevented the expansion of other fish species. The later, advanced phase of the improvement suppressed Prussian carp and weatherfish, but enabled the expansion of numerous native species, mainly ide. The drivers of both the initial and advanced phases were increasing dissolved oxygen content, and decreasing BOD_5_, total phosphorus and perhaps calcium content. The advanced phase of the recovery was probably supported by an ecomorphological interaction between Prussian carp and native ide.

## Supplementary Information

Supplementary Information.

## References

[1] Petsch DK (2016). Causes and consequences of biotic homogenization of freshwater ecosystems. Int. Rev. Hydrobiol..

[2] Chen GZ, Qiu YP, Li LP (2017). Fish invasions and changes in the fish fauna of the Tarim Basin. Acta Ecol. Sin..

[3] Rau MA, Plavan G, Strungaru SA, Nicoara M, Rodriguez-Lozano P, Mihu-Pintilie A (2017). The impact of amur sleeper (*Percottus glenii* Dybowsky 1877) on the riverine ecosystem: Food selectivity of amur sleeper in a recently colonized river. Oceanol. Hydrobiol. Stud..

[4] FishBase, *Carassius gibelio* Bloch 1972, http://fishbase.org (2017).

[5] Olden JD, Comte L, Giam X (2016). Biotic Homogenisation. eLS.

[6] Savini D, Occhipinti-Ambrogi A, Marchini A, Tricarico E, Gherardi F, Olenin S (2010). The top 27 animal alien species introduced into Europe for aquaculture and related activities. J. Appl. Ichthyol..

[7] Elgin EL, Tunna HR, Jackson LJ (2014). First confirmed records of Prussian carp *Carassius gibelio* (Bloch 1782) in open waters of North America. BioInvasions Rec..

[8] Özdilek ŞY, Jones RI (2014). The diet composition and trophic position of introduced Prussian carp *Carassius gibelio* (Bloch 1782) and native fish species in a Turkish river. Turk. J. Fish. Aquat. Sci..

[9] Docherty, C. Establishment, spread and impact of Prussian carp (*Carassius gibelio*), a new invasive species in Western North America. (Master of Science thesis, Alberta, Canada, University of Alberta, 2016).

[10] Leonardos ID, Tsikliras AC, Eleftheriou V, Cladas Y, Kagalou I, Chortatou R (2008). Life history characteristics of an invasive cyprinid fish (*Carassius gibelio*) in Chimaditis Lake (northern Greece). J. Appl. Ichthyol..

[11] Papoušek I, Vetešník L, Halačka K, Lusková V, Humpl M, Mendel J (2008). Identification of natural hybrids of gibel carp *Carassius**auratus**gibelio* (Bloch) and crucian carp *Carassius**carassius* (L.) from lower Dyje River floodplain (Czech Republic). J. Fish Biol..

[12] Lusková V, Lusk S, Halačka K, Vetešník L (2010). *Carassius auratus gibelio*—the most successful invasive fish in waters of the Czech Republic. Russ. J. Biol. Invasions.

[13] Specziár A, Tölg L, Bíró P (1997). Feeding strategy and growth of cyprinids in the littoral zone of Lake Balaton. J. Fish Biol..

[14] Slavík O, Bartoš L (2004). What are the reasons for the Prussian carp expansion in the upper Elbe River Czech Republic?. J. Fish Biol..

[15] Eyckmans M, Celis N, Horemans N, Blust R, De Boeck G (2011). Exposure to waterborne copper reveals differences in oxidative stress response in three freshwater fish species. Aquat. Toxicol..

[16] Liasko R, Koulish A, Pogrebniak A, Papiggioti O, Taranenko L, Leonardos I (2011). Influence of environmental parameters on growth pattern and population structure of *Carassius auratus gibelio* in Eastern Ukraine. Hydrobiologia.

[17] Rylková K, Kalous L, Bohlen J, Lamatsch DK, Petrtýl M (2013). Phylogeny and biogeographical history of the cyprinid fish genus *Carassius* (Teleostei: Cyprinidae) with focus on natural and anthropogenic arrivals in Europe. Aquaculture.

[18] Daněk T, Kalous L, Veselý T, Krásová E, Reschová S, Rylková K (2012). Massive mortality of Prussian carp *Carassius gibelio* in the upper Elbe basin associated with herpesviral hematopoietic necrosis (CyHV-2). Dis. Aquat. Org..

[19] Xu J, Zeng L, Zhang H, Zhou Y, Ma J, Fan Y (2013). Cyprinid herpesvirus 2 infection emerged in cultured gibel carp *Carassius auratus gibelio* in China. Vet. Microbiol..

[20] Nanjo A, Shibata T, Saito M, Yoshii K, Tanaka M, Nakanishi T (2017). Susceptibility of isogeneic ginbuna *Carassius auratus langsdorfii* Temminck et Schlegel to cyprinid herpesvirus-2 (CyHV-2) as a model species. J. Fish Dis..

[21] Ferincz Á, Horváth Z, Staszny Á, Ács A, Kováts N, Vad CF (2016). Desiccation frequency drives local invasions of non-native gibel carp (*Carassius gibelio*) in the catchment of a large shallow lake (Lake Balaton Hungary). Fish. Res..

[22] Kulmatycki, W. Hydrografia i rybostan rzek województwa łódzkiego. *Czasopismo Przyrodnicze Ilustrowane* **10**, 123–150 **(in Polish)** (1936).

[23] Penczak T (1975). Ichthyofauna of the catchment area of the River Ner and perspectives of its restitution in connection with the erection of a collective sewage treatment plant for the Agglomeration of the City of Łódź. Acta Hydrobiol..

[24] Penczak T, Głowacki Ł, Kruk A (2017). Fish recolonization of a lowland river with non-buffered storm water discharges but with abated pollution from a large municipality. Ecol. Ind..

[25] Alabaster JS, Lloyd R (1984). Water Quality Criteria for Freshwater Fish.

[26] Matthews WJ (2012). Patterns in Freshwater Fish Ecology.

[27] Baltz M, Moyle PB (1993). Invasion resistance to introduced species by a native assemblage of California stream fishes. Ecol. Appl..

[28] Carlsson NOL, Bustamante H, Strayer DL, Pace ML (2011). Biotic resistance on the increase: Native predators structure invasive zebra mussel populations. Freshw. Biol..

[29] Ter Braak, C. J. F. & Smilauer, P. CANOCO reference manual and user’s guide to Canoco for Windows—software for canonical community ordination (version 4). (Microcomputer Power, 1998).

[30] Legendre P, Gallager E (2001). Ecologically meaningful transformations for ordination of species data. Oecologia.

[31] Sokal RR, Rohlf FJ (1995). Biometry: The Principles and Practice of Statistics in Biological Research.

[32] Legendre P, Legendre L (2012). Numerical Ecology.

[33] Legendre, P. Model II regression user’s guide R edition. http://137.132.33.20/web/packages/lmodel2/vignettes/mod2user.pdf (2013, accessed 2017).

[34] Legendre, P. Model II regression—User’s guide. http://adn.biol.umontreal.ca/~numericalecology/download,html#ModelIIregression (2001, accessed 2017).

[35] Holm S (1979). A simple sequentially rejective multiple test procedure. Scand. J. Stat..

[36] Bohlin T, Hamrin S, Heggberget TG (1989). Electrofishing—Theory and practice with special emphasis on salmonids. Hydrobiologia.

[37] De Boeck G, Smolders R, Blust R (2010). Copper toxicity in gibel carp *Carassius auratus gibelio*: Importance of sodium and glycogen. Comp. Biochem. Physiol. C.

[38] Gui JF, Zhou L (2010). Genetic basis and breeding application of clonal diversity and dual reproduction modes in polyploid *Carassius auratus gibelio*. Sci. China Life Sci..

[39] Pyrzanowski K, Zięba G, Dukowska M, Smith C, Przybylski M (2019). The role of detritivory as a feeding tactic in a harsh environment—A case study of weatherfish (*Misgurnus fossilis*). Sci. Rep..

[40] Meyer L, Hinrichs D (2000). Microhabitat preferences and movements of the weatherfish, *Misgurnus fossilis*, in a drainage channel. Environ. Biol. Fish..

[41] Paulovits G, Ferincz Á, Staszny Á, Weiperth Á, Tátrai I, Korponai J (2014). Long-term changes in the fish assemblage structure of a shallow eutrophic reservoir (Lake Hídvégi Hungary) with special reference to the exotic *Carassius gibelio*. Int. Rev. Hydrobiol..

[42] Schiemer F, Wieser W (1992). Epilogue: Food and feeding ecomorphology energy assimilation and conversion in cyprinids. Environ. Biol. Fish..

[43] Rohtla M, Vilizzi L, Kováč V, Almeida D, Brewster B, Britton JR (2020). A stranger in our midst—Review and meta-analysis of the environmental biology and potential invasiveness of a poorly studied European cyprinid, the ide *Leuciscus**idus*. Rev. Fish. Sci. Aquac..

[44] Tadajewska, M. Jaź [ide] *Leuciscus idus* (Linnaeus 1758) in Ryby słodkowodne Polski [Freshwater fish of Poland] (in Polish). (ed. Brylińska, M.) 314–318 (PWN, 2000).

[45] Eklöv AG, Greenberg LA, Brönmark C, Larsson P, Berglund O (1998). Response of stream fish to improved water quality: A comparison between the 1960s and 1990s. Freshw. Biol..

[46] Kruk A (2007). Role of habitat degradation in determining fish distribution and abundance along the lowland Warta River Poland. J. Appl. Ichthyol..

[47] Przybylski M (1993). Longitudinal pattern in fish assemblages in the upper Warta River Poland. Arch. Hydrobiol..

[48] Kruk A, Ciepłucha M, Zięba G, Błońska D, Marszał L, Tybulczuk S (2017). Disturbed fish fauna zonation as an indicator of large-scale human impact: A case study (2011–2012) of the large lowland Warta River Poland. J. Appl. Ichthyol..

[49] Kruk A, Penczak T (2003). Impoundment impact on populations of facultative riverine fish. Ann. Limnol. Int. J. Limnol..

[50] Głowacki Ł (2012). Accuracy of species richness estimators applied to fish in small and large temperate lowland rivers. Biodivers. Conserv..

[51] Głowacki Ł, Penczak T (2012). Large dam reservoirs are probably long period oscillators of fish diversity. J. Fish Biol..

[52] Penczak T, Głowacki Ł, Kruk A, Galicka W (2012). Implementation of a self-organizing map for investigation of impoundment impact on fish assemblages in a large lowland river: Long-term study. Ecol. Model..

[53] Ciepłucha, M., Kruk, A., Zięba, G., Marszał, L., Tszydel, M., Tybulczuk, S. *et al.* Fish fauna of the Warta River. *Scientific Annual of the Polish Angling Association* **27**, 147–184 **(in Polish with English summ.)** (2014).

[54] Tybulczuk, S., Marszał, L., Kruk., Janic, B., Pietraszewski, D., Błońska, D. *et al.* Fish fauna of the Gwda River system (2013–2015). *Scientific Annual of the Polish Angling Association* **30**, 59–94 **(in Polish with English summ.)** (2017).

[55] Kruk A, Penczak T (2013). Natural regeneration of fish assemblages in the Pilica River after reduction of point source pollution. River Res. Appl..

[56] Głowacki Ł, Penczak T (2013). Drivers of fish diversity homogenization/differentiation and species range expansions at the watershed scale. Divers. Distrib..

[57] Ricklefs RE (1987). Community diversity: Relative roles of local and regional processes. Science.

[58] Cornell HV, Lawton JH (1992). Species interactions local and regional processes and limits to the richness of ecological communities: A theoretical perspective. J. Anim. Ecol..

[59] Bøhn T, Sandlund OT, Amundsen P-A, Primicerio R (2004). Rapidly changing life history during invasion. Oikos.

[60] Kong SY, Jiang YS, Wang Q, Lu JF, Xu D, Lu LQ (2017). Detection methods of Cyprinid herpesvirus 2 infection in silver crucian carp (*Carassius auratus gibelio*) via a pORF72 monoclonal antibody. J. Fish Dis..

[61] Górski K, Buijse AD, Winter HV, De Leeuw JJ, Compton TJ, Vekhov DA (2013). Geomorphology and flooding shape fish distribution in a large-scale temperate floodplain. River Res. Appl..

[62] Tilman D (1982). Resource Competition and Community Structure.

[63] Shea K, Chesson P (2002). Community ecology theory as a framework for biological invasions. Trends Ecol. Evol..

[64] Elton CS (1958). The Ecology of Invasions by Animals and Plants.

[65] Harvey BC, White JL, Nakamoto RJ (2004). An emergent multiple predator effect may enhance biotic resistance in a stream fish assemblage. Ecology.

[66] Facon B, Genton BJ, Shykoff J, Jarne P, Estoup A, David P (2006). A general eco-evolutionary framework for understanding bioinvasions. Trends Ecol. Evol..

[67] Balon EK (1975). Reproductive guilds of fishes: A proposal and definition. J. Fish. Res. Board Can..

[68] Simberloff D, Von Holle B (1999). Positive interactions of nonindigenous species: Invasional meltdown?. Biol. Invasions.

[69] Poff NL (1997). Landscape filters and species traits: Towards mechanistic understanding and prediction in stream ecology. J. N. Am. Benthol. Soc..

