# Supplementary material for: Advancing improvement in riverine water quality caused a non-native fish species invasion and native fish fauna recovery
Source: Sci Rep. 2021 Aug 13;11:16493. doi: 10.1038/s41598-021-93751-2 (PMC8363613; doi:10.1038/s41598-021-93751-2)
Supplement: Supplementary file 1 — Supplementary Information. [file 41598_2021_93751_MOESM1_ESM.pdf]

Appendix. Supplementary material/information

(Table S1, Table S2, Table S3, Figure S1)

to the study:

"Advancing improvement in riverine water quality caused a non-native fish species invasion and native fish fauna recovery"

by

Łukasz Głowacki, Andrzej Kruk, Tadeusz Penczak

published in the journal of **Scientific Reports** in 2021

DOI: 10.1038/s41598-021-93751-2

Species / year of sampling	2000	2002	2004	2005	2008	2010	2012	Total
Alburnus alburnus – bleak	0	77	6	1	168	171	547	970
Blicca bjoerkna – silver bream	0	637	193	1349	288	153	2660	5280
Carassius carassius – crucian carp	0	56	0	0	24	77	0	157
Carassius gibelio – Prussian carp	4897	7316	1135	2142	199	2918	13	18620
Cobitis taenia – spined loach	0	34	6	29	22	24	14	129
Esox lucius – pike	0	12797	4591	6584	6662	18078	2814	51526
Gasterosteus aculeatus – stickleback	2	62	6	0	19	1	1	91
Gogio gobio – gudgeon	0	7	1519	1175	799	288	1278	5066
Gymnocephalus cernua – ruffe	0	145	18	78	12	36	54	343
Leuciscus idus – ide	0	620	1090	7064	14360	5796	10693	39623
Lota lota – burbot	0	263	0	1882	1310	1542	662	5659
Misgurnus fossilis – weatherfish	3332	5115	0	186	33	6	48	8720
Perca fluviatilis – perch	4	5777	3383	6345	5866	3979	6029	31383
Rutilus rutilus – roach	25	2621	2877	16425	9472	8661	7642	47723
Sander lucioperca – pikeperch	0	0	519	0	65	13	105	702
Tinca tinca – tench	0	67	1758	997	10635	2701	885	17043
Total	8260	35594	17101	44257	49934	44444	33445	233035

Table S1. Complete raw (untransformed) biomass (in grams recalculated per 500 m of bankline) data set of those fish species sampled in the recovered course of the Ner River, Poland, in 2000-2012 for which regression models presented in this study could be calculated. Species are ordered according to their Latin generic names.

Fish species (scientific name – common name)	R^2_{adj}	Slope	Intercept	$F_{[1,5]}$	*P*-value	Holm adjusted *P*-value
Leuciscus idus – ide	0.913	-1.18	0.67	63.5931	0.0005	0.0070
Misgurnus fossilis – mud loach	0.807	0.95	0.14	26.0193	0.0038	0.0494
Rutilus rutilus – roach	0.688	-1.24	0.77	14.2351	0.0130	0.1560
Perca fluviatilis – perch	0.513	-1.35	0.74	7.3132	0.0426	0.4686
Tinca tinca – tench	0.401	-1.13	0.51	5.0100	0.0754	0.7540
Gobio gobio – gudgeon	0.308	-1.82	0.51	3.6701	0.1135	1.0000
Alburnus alburnus – bleak	0.307	-1.57	0.49	3.6589	0.1140	1.0000
Blicca bjoerkna – silver bream	0.303	-3.74	0.46	3.6106	0.1158	1.0000
Lota lota – burbot	0.284	-1.90	0.50	3.3774	0.1255	1.0000
Cobitis taenia – spined loach	0.206	-15.28	0.60	2.5612	0.1704	1.0000
Esox lucius – pike	-0.009	-0.47	0.48	0.9438	0.3759	1.0000
Gasterosteus aculeatus – stickleback	-0.053	6.38	0.20	0.6992	0.4412	1.0000
Sander lucioperca – pikeperch	-0.083	-1.27	0.34	0.5427	0.4944	1.0000
Gymnocephalus cernua – ruffe	-0.095	-3.69	0.41	0.4792	0.5196	1.0000

Table S2. OLS model I regressions used to assess the dependence of *Carassius gibelio* – Prussian carp biomass on the biomass of other fish species that were sampled in the recovered course of the Ner River, Poland, over the period of 2000-2012. Number of cases (i.e. of sampling surveys) was 7 in all models. If model I regression (OLS method using Hellinger transformed biomass data) were used instead of MA regression (with Hellinger-transformed data), the number and sequence of significant models would be slightly different: four models would be significant before Holm[35] adjustment, and two after the adjustment. Besides, the weatherfish vs. Prussian carp model instead of the roach vs. Prussian carp one would be the second most important. Also, significance differences between OLS models would be greater than those between MA ones. MA regression applied to the other initial (at the beginning of the sampling period) dominant fish species in the recovered course, weatherfish, was not related to the biomass changes of any other fish species (after Holm[35] adjustment) (Table S3). Fig. S1 shows how a decreased significance of an MA model may affect a respective graph (here of roach biomass vs. Prussian carp biomass): the confidence limit lines are much wider apart, and the shape of the ellipse is much less elongated than that of the best (i.e. ide vs. Prussian carp) model.

Fish species (scientific names – common names)	Eigenvalues		H statistic	Minor Axis		Major Axis						95% Confidence limits of intercept of major axis		95% Confidence limits of slope of major axis	
	λ1	λ2		Intercept	Slope	Intercept	Slope	Angle (degrees)	*P*-value (1-tailed)	Holm adjusted *P*-value		2.5% - Intercept	97.5% - Intercept	2.5% - Slope	97.5% - Slope
Tinca tinca – tench	0.078	0.008	0.172	0.051	0.577	0.508	-1.734	-60.021	0.0040	0.0560		0.306	2.239	-0.713	-10.480
Gobio gobio – gudgeon	0.067	0.004	0.095	0.121	0.347	0.526	-2.880	-70.855	0.0107	0.1391		6.306	0.331	-48.986	-1.321
Sander lucioperca – pikeperch	0.061	0.003	0.072	0.160	0.126	0.486	-7.935	-82.818	0.0107	0.1391	‡	-0.110	0.262	6.802	-2.387
Carassius gibelio – Prussian carp	0.120	0.005	0.063	0.467	-1.038	-0.116	0.964	43.942	0.0171	0.1881		0.001	-0.311	0.563	1.634
Leuciscus idus – ide	0.095	0.008	0.138	-0.096	0.814	0.559	-1.229	-50.863	0.0192	0.1920		1.193	0.343	-3.203	-0.556
Rutilus rutilus – roach	0.087	0.004	0.071	-0.102	0.692	0.723	-1.446	-55.331	0.0236	0.2124		1.271	0.487	-2.867	-0.836
Perca fluviatilis – perch	0.074	0.007	0.159	-0.003	0.507	0.817	-1.973	-63.119	0.0827	0.6616		5.786	0.439	-16.992	-0.827
Lota lota – burbot	0.063	0.005	0.116	0.141	0.213	0.690	-4.704	-77.998	0.0921	0.6616	‡	-0.641	0.344	7.218	-1.607
Esox lucius – pike	0.085	0.023	0.679	-0.157	0.805	0.661	-1.242	-51.160	0.1220	0.7320		-1.171	0.135	3.342	0.076
Blicca bjoerkna – silver bream	0.063	0.006	0.166	0.142	0.190	0.791	-5.273	-79.261	0.1359	0.7320	‡	-0.338	0.336	4.234	-1.441
Cobitis taenia – spined loach	0.061	0.000	0.001	0.164	0.022	1.060	-44.767	-88.720	0.1417	0.7320	‡	-1.165	0.499	66.499	-16.729
Alburnus alburnus – bleak	0.061	0.002	0.036	0.161	0.078	0.751	-12.899	-85.567	0.1556	0.7320	‡	-0.230	0.330	8.693	-3.623
Gasterosteus aculeatus – stickleback	0.061	0.000	0.003	0.165	-0.025	-0.441	40.064	88.570	0.2113	0.7320	‡	-0.015	0.611	11.906	-29.502
Gymnocephalus cernua – ruffe	0.061	0.000	0.009	0.164	0.021	1.697	-48.214	-88.812	0.2833	0.7320	‡	-0.275	0.443	13.837	-8.763

Table S3. Major Axis (MA) model II regressions used to assess the dependence of *Misgurnus fossilis* – weatherfish biomass on the biomass of other fish species that were sampled in the recovered course of the Ner River, Poland, over the period of 2000-2012. Number of cases (i.e. of sampling surveys) was 7 in all models; ‡ – monotonicity adjustment.

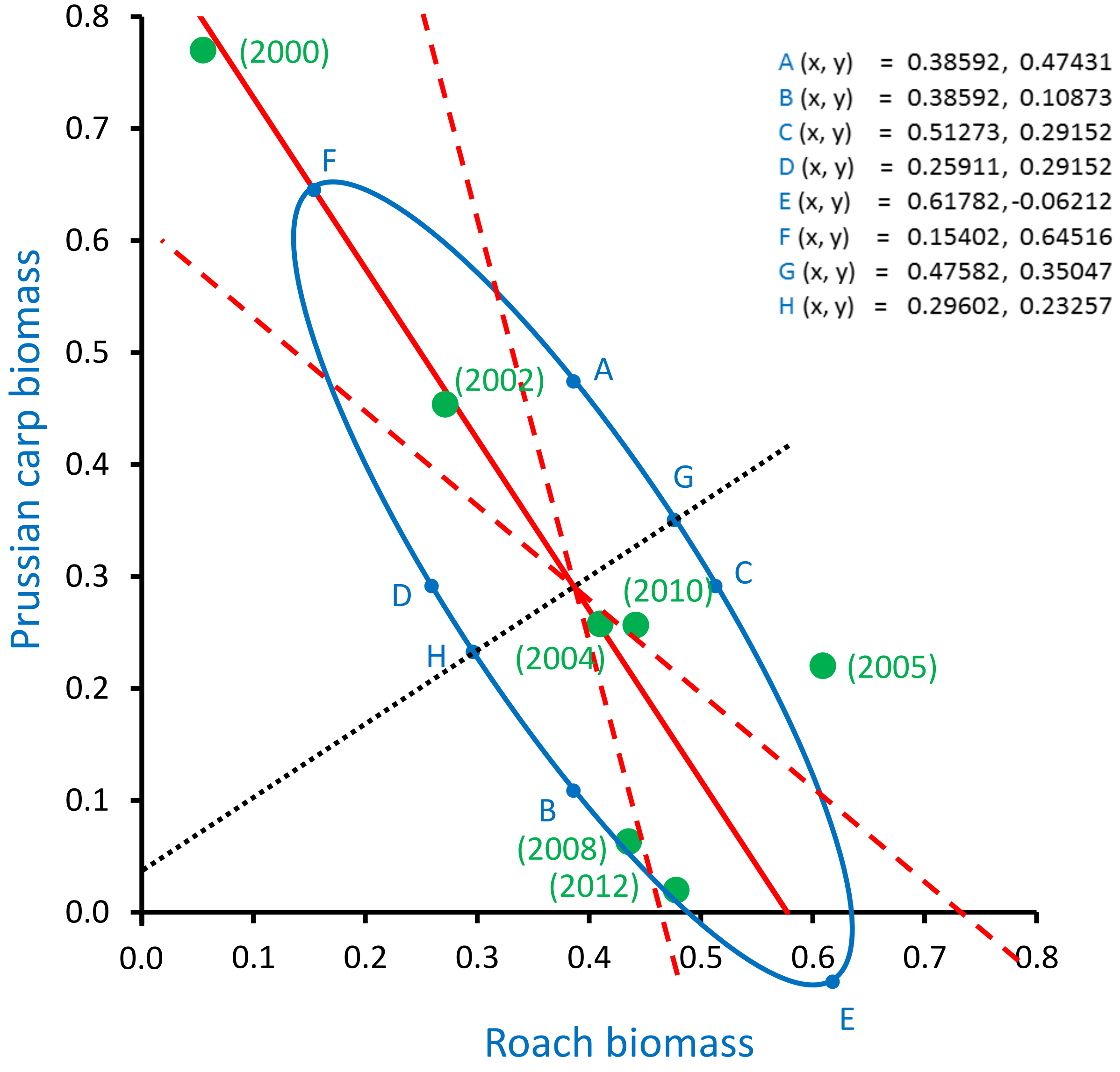

Figure S1. Prussian carp biomass as a function of roach biomass (both Hellinger transformed) in the recovered course of the Ner River, Poland, in subsequent sampling surveys over the period of 2000-2012. Green dots are biomass values in given surveys, while green numbers in brackets are years of the surveys. Red solid line is the main axis (of the bivariate distribution and of the ellipse). Red dashed lines are 95% confidence limits of the major axis. Black dotted line is the minor axis. The blue ellipse is the 95% confidence region of the mean of both variables. Blue dots (marked by blue letters, 'A…H'), are characteristic points of the ellipse. The coordinates of the points (e.g. 'A (x, y) =') are also indicated, x being the abscissa, y the ordinate axis location.
